# Tumor metabolism and associated serum metabolites define prognostic subtypes of Asian hepatocellular carcinoma

**DOI:** 10.1038/s41598-021-91560-1

**Published:** 2021-06-08

**Authors:** Yotsawat Pomyen, Anuradha Budhu, Jittiporn Chaisaingmongkol, Marshonna Forgues, Hien Dang, Mathuros Ruchirawat, Chulabhorn Mahidol, Xin Wei Wang, the TIGER-LC Consortium, Benjarath Pupacdi, Siritida Rabibhadana, Kannikar Phonphutkul, Nirush Lertprasertsuke, Anon Chotirosniramit, Chirayu U. Auewarakul, Teerapat Ungtrakul, Vajarabhongsa Budhisawasdi, Chawalit Pairojkul, Suleeporn Sangrajang, Curtis C. Harris, Christopher A. Loffredo, Robert Wiltrout

**Affiliations:** 1 Laboratory of Human Carcinogenesis, Center for Cancer Research, National Cancer Institute, Bethesda, MD 20892 USA; 2 Translational Research Unit, Chulabhorn Research Institute, Bangkok, 10210 Thailand; 3 Liver Cancer Program, Center for Cancer Research, National Cancer Institute, Bethesda, MD 20892 USA; 4 Laboratory of Chemical Carcinogenesis, Chulabhorn Research Institute, Bangkok, 10210 Thailand; 5 Center of Excellence on Environmental Health and Toxicology, Office of Higher Education Commission, Ministry of Higher Education, Science, Research and Innovation, Bangkok, 10400 Thailand; 6 Present Address: Division of Surgery, Thomas Jefferson University, Philadelphia, PA 19107 USA; 7 Rajavej Hospital, Chiang Mai, 50000 Thailand; 8 Faculty of Medicine, Chiang Mai University, Chiang Mai, 50200 Thailand; 9 Chulabhorn Royal Academy, Bangkok, 10210 Thailand; 10 Faculty of Medicine, Khon Kaen University, Khon Kaen, 40002 Thailand; 11 National Cancer Institute, Bangkok, 10400 Thailand; 12 Georgetown University Medical Center, Washington, DC 20057 USA; 13 Center for Cancer Research, National Cancer Institute, Bethesda, MD 20892 USA

**Keywords:** Hepatocellular carcinoma, Transcriptomics, Metabolomics

## Abstract

Treatment effectiveness in hepatocellular carcinoma (HCC) depends on early detection and precision-medicine-based patient stratification for targeted therapies. However, the lack of robust biomarkers, particularly a non-invasive diagnostic tool, precludes significant improvement of clinical outcomes for HCC patients. Serum metabolites are one of the best non-invasive means for determining patient prognosis, as they are stable end-products of biochemical processes in human body. In this study, we aimed to identify prognostic serum metabolites in HCC. To determine serum metabolites that were relevant and representative of the tissue status, we performed a two-step correlation analysis to first determine associations between metabolic genes and tissue metabolites, and second, between tissue metabolites and serum metabolites among 49 HCC patients, which were then validated in 408 additional Asian HCC patients with mixed etiologies. We found that certain metabolic genes, tissue metabolites and serum metabolites can independently stratify HCC patients into prognostic subgroups, which are consistent across these different data types and our previous findings. The metabolic subtypes are associated with β-oxidation process in fatty acid metabolism, where patients with worse survival outcome have dysregulated fatty acid metabolism. These serum metabolites may be used as non-invasive biomarkers to define prognostic tumor molecular subtypes for HCC.

## Introduction

Hepatocellular carcinoma (HCC) is one of the leading cancers worldwide in both incidence and mortality. According to the Global Cancer Observatory, the number of new liver cancer cases worldwide is 4.7% of all cancer incidence but accounted for 8.2% of all cancer deaths in 2018^1^. It is estimated that there will be a 64% increase in incidence and a 69% increase in mortality of liver cancer by 2040 based on 2018 statistics^2^. Most of these incidence and deaths will occur in East Asia and Southeast Asia, including Thailand.

In the past, Thailand had one of the highest incidence of HCC due to the high prevalence of hepatitis B virus (HBV) infection^3^ and food contamination by aflatoxin B_1_ (AFB_1_)^4,5^. However, lifestyle changes and economic improvement over several decades have shifted the cause of HCC from HBV infection and food contamination to new causes, such as Hepatitis C virus (HCV) infection^6^ and non-alcoholic fatty liver disease (NAFLD)^7,8^. The shift in the landscape of hepatocarcinogenesis is now a challenge for healthcare professionals. Currently, α-fetoprotein (AFP) is the most convenient and non-invasive serum biomarker for detecting HCC^9^. However, the reliability of AFP compared to ultrasound is still an ongoing debate for early-stage detection of the HCC^10,11^. Therefore, there is an urgent need for better serum biomarkers to achieve early detection and better patient stratification for precision oncology to improve patient prognosis^9,10^.

Previously, our group has shown that serum AFP alone is not sufficient as a biomarker for early detection or patient stratification of aggressive HCC subtypes in Asian patient cohorts^12,13^. With the shortcomings of AFP in Asian populations, we have established a gene-signature panel of molecular subtypes that can consistently stratify aggressive HCC and intrahepatic cholangiocarcinoma (iCCA) subtypes among several Asian populations^13^. However, this and previous signatures, are mainly based on gene expression and miRNA expression signatures derived from bulk tumor tissues, which are difficult to obtain without surgical resection of the tumors^14^. In this study, we reasoned that the molecular subtype-related tumor biology may have a systemic impact and, therefore, be reflected through the systemic serum metabolite profiles of the patients. To identify serum metabolites that are surrogates of gene-based molecular subtypes, we employed metabolite-focused, two-step correlation analysis, first with correlation analysis between tissue gene expression of metabolic genes and tissue metabolite profiles, and second, between tissue metabolite profiles and serum metabolite profiles. This strategy allowed us to identify serum metabolite panels that can stratify HCC patients according to their tumor-related prognostic subtypes.

## Results

### Tissue metabolites can distinguish between tumor and non-tumor tissue

**Figure 1:**
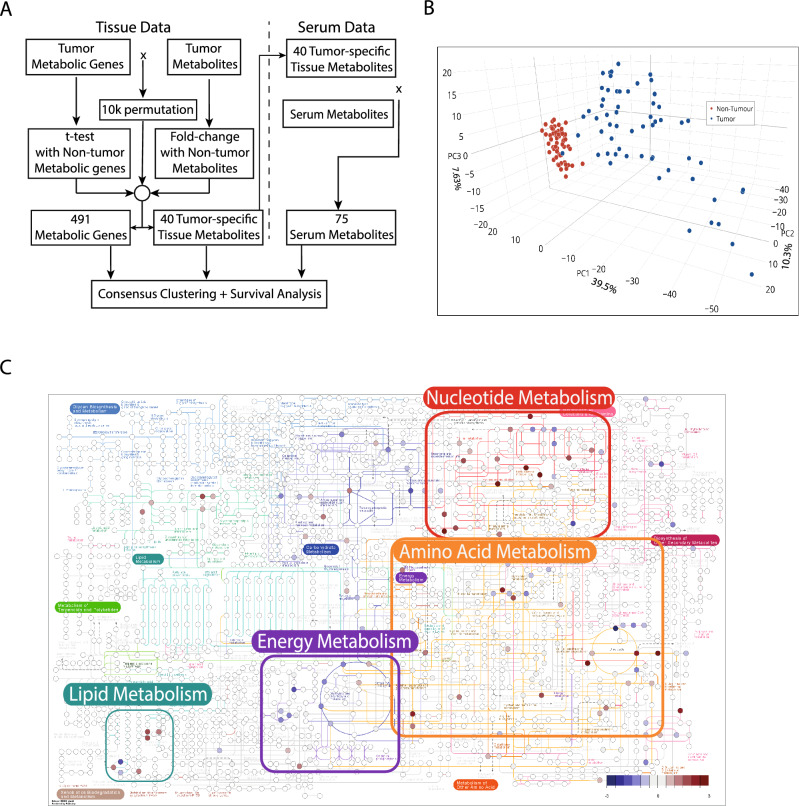
Overall study design, tissue stratification by metabolite abundance and their related pathways. (**A**) Overall flowchart of the analysis. (**B**) 3D PCA plot of tumor and adjacent non-tumor tissue metabolites from Thai HCC patients based on expression of 500 most variable metabolites. The tumor and non-tumor tissues can be clearly separated by tissue metabolites alone. (**C**) KEGG Overall Human Pathways (map accession number hsa01100^15^) overlaid with metabolite ratios between tumor and adjacent non-tumor tissues from HCC patients. Each node represents a human metabolite in a metabolic pathway. The colors represent log2 fold-change of the tissue metabolites. Blue represents increased mean metabolite abundance from adjacent non-tumor tissues, and red represents higher mean metabolite abundance from tumor tissues. White nodes represent metabolites with no data available. Abbreviations: PCA—Principal Component Analysis; HCC—Hepatocellular Carcinoma.

The overall flowchart of the analysis strategy is illustrated in Fig. 1A, with a detailed analysis flowchart shown in Supplementary Fig. S1. To test whether tissue metabolites can distinguish tumor and non-tumor tissues of 56 HCC patients, Principal Component Analysis (PCA) was used to classify tissue samples based on metabolites measured by an untargeted metabolomics approach (Fig. 1B). Based on the 500 metabolites with highest variability among samples, tumor and non-tumor tissues can be separated clearly. To visualize the extent of tissue metabolites and pathways that are dysregulated, differentially altered metabolites between tumor and non-tumor tissues were overlaid onto overall human metabolite map from KEGG^15^. The 562 known detected tissue metabolites can be converted to 352 KEGG compound IDs from the total of 1271 metabolites present on the map (accession number hsa01100). The affected metabolites in HCC fall into four main metabolic categories, i.e., nucleotide metabolism, amino acid metabolism, energy metabolism, and lipid metabolism (Fig. 1C).

### Metabolic genes, tissue and serum metabolites can identify patients with worse survival outcome

**Figure 2:**
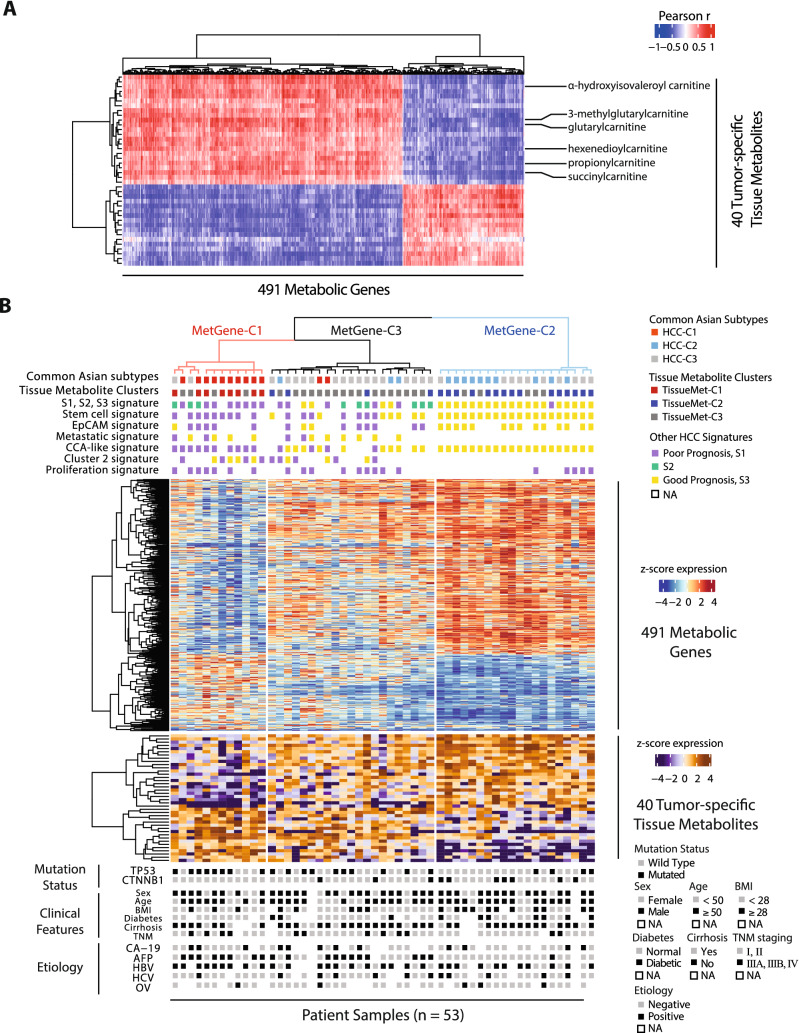
Correlation analysis of tumor metabolic genes and tissue metabolites. (**A**) Heatmap of correlation coefficients between 491 metabolic genes and 40 tumor-specific tissue metabolites that pass the thresholds of 0.165 and − 0.165 defined in by permutation test. (**B**) Heatmap of gene expression from 491 metabolic genes in the tumor tissues from HCC patients from TIGER-LC cohort. The top panel of the heatmap shows clusters of patients based on three classifications of patients: (1) the dendrogram is based on consensus clustering from 491 metabolic genes identified through permutation test (MetGene clusters); (2) common Asian subtypes identified previously by our research group^13^; and (3) Tissue Metabolite (TissueMet) clusters, which are based on consensus clustering from 40 tumor-specific tissue metabolites. The middle panel shows two heatmaps based on z-score expression of 491 metabolic genes and 40 tumor-specific tissue metabolites. The bottom panel of the heatmap shows mutation status of two genes (TP53 and CTNNB1), clinical features, and etiologies of each tissue sample. Abbreviations: MetGene-C—Metabolic gene-Cluster; TissueMet-C—Tissue metabolite-Cluster; HCC—Hepatocellular Carcinoma; BMI—Body Mass Index; TNM—TNM Classification of Malignant Tumors; CA-19—Cancer Antigen 19–9; AFP—Alpha Fetoprotein; HBV—Hepatitis B virus; HCV—Hepatitis C virus; OV—*Opisthorchis viverrini.* Each colored box above heatmap represents one sample. The color representing “Good” prognosis and S3 signature is yellow, while “Poor” prognosis and S1 signature is represented by purple. S2 signature is represented by green box.

**Figure 3:**
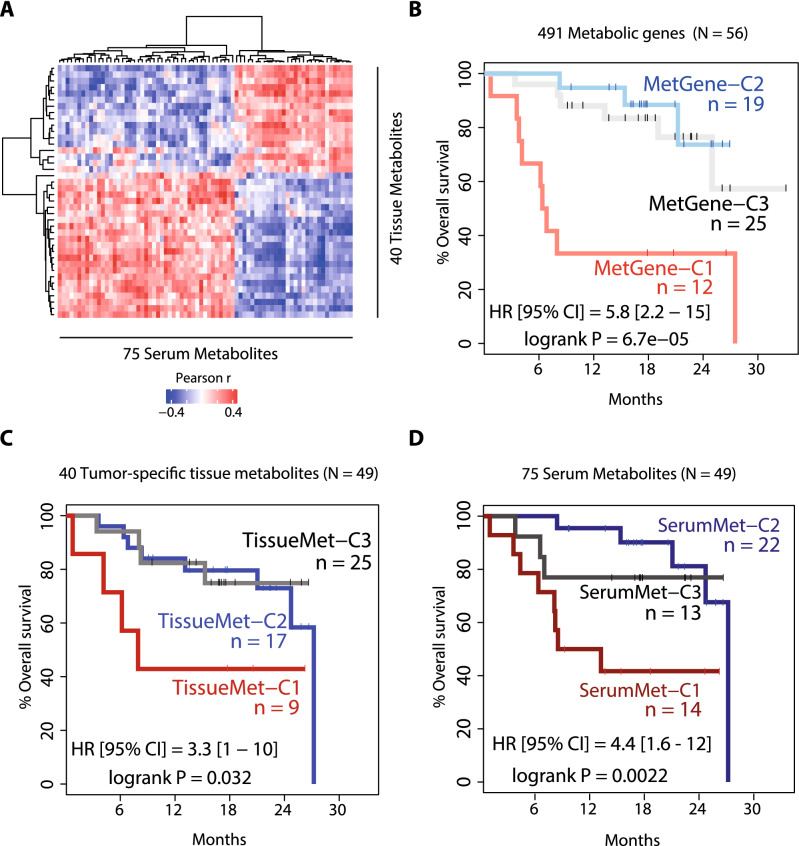
Correlation analysis of tissue and serum metabolites. (**A**) Heatmap of correlation coefficients between 40 tumor-specific tissue metabolites and 75 serum metabolites identified in this study. (**B**) Kaplan–Meier plot showing survival probabilities of HCC patients from TIGER-LC cohort according to consensus clustering based on expression of 491 metabolic genes. (**C**) Kaplan–Meier plot showing survival probabilities of HCC patients from TIGER-LC cohort according to consensus clustering based on abundance of 40 tumor-specific metabolites. (**D**) Kaplan–Meier plot showing survival probabilities of HCC patients from TIGER-LC cohort according to consensus clustering based on abundance of 75 serum metabolites. Abbreviations: HR [95% CI]—Hazard Ratio with 95% confidence interval; MetGene-C—Metabolic gene-Cluster; TissueMet-C—Tissue metabolite-Cluster; SerumMet-C—Serum metabolite-Cluster.

We used metabolism-specific genes to further determine if they can classify HCC into prognostic subtypes. To ensure that the metabolic genes and tissue metabolites are tumor-specific, we incorporated the calculation of a paired t-test between tumor and adjacent non-tumor tissue gene expression. For the first step of the correlation analysis, the numbers of metabolic genes and metabolites that passed correlation thresholds from a permutation test and a paired t-test with an FDR-adjusted *p*-value of < 0.05 were 491 genes and 78 metabolites, respectively (Supplementary Table S1). Among 78 metabolites that passed the correlation coefficient thresholds (Supplementary Table S2), 40 metabolites passed an additional 1.5 fold-change cutoff between tumor and adjacent non-tumor tissues (Fig. 2A, Supplementary Fig. S2A, and Supplementary Table S3). We performed hierarchical clustering analysis on Thai HCC patient samples by using consensus clustering algorithm^16^ based on these 491 metabolic genes (MetGene) or 40 tumor-specific metabolites (TissueMet) which revealed three stable clusters based on metabolic genes or tumor-specific tissue metabolites, named MetGene-C1, MetGene-C2, and MetGene-C3, or TissueMet-C1, TissueMet-C2, and TissueMet-C3, respectively (Fig. 2B—upper panel). The metabolic gene expression based on 491 genes shows distinct expression patterns between the three clusters, which are similar to previously identified common Asian subtypes (Fig. 2B—middle panel)^13^. Similar to global transcriptome profiling analysis, there was no discernible pattern emerging from standard clinical and etiological parameters of HCC (Fig. 2B—lower panel). These results suggest that metabolic genes can capture major molecular features of tumor samples. We also explored whether these subclasses were consistent with prognostic signatures published among HCC cohorts. To do so, we assessed S1-S3 signature^17^, stem cell signature^18^, EpCAM signature^19^, Metastatic signature^20^, CCA-like signature^21^, Cluster 2 signature^22^, proliferation signature^23^ and found a concordance between the good and poor survival subclasses defined by the metabolic genes and those defined by published signatures. We also performed consensus clustering using the same set of metabolic genes in two other cohorts: Liver Cancer Institute (LCI)^20^ cohort and The Cancer Genome Atlas (TCGA)^24^ cohort on Asian patients only. Similar gene expression patterns were observed in both cohorts (Supplementary Fig. S3A, B). The resulting MetGene clusters from LCI and TCGA cohorts were then subjected to Subclass Mapping analysis to identify clusters that matched the original consensus clusters from the TIGER-LC cohort. MetGene-C1 and MetGene-C2 clusters matched the original clusters from TIGER-LC cohort (Supplementary Fig. S3C, D). To identify serum metabolites that are directly associated with tumor tissue metabolites, we performed a second step of correlation analysis between the 40 tumor-specific tissue metabolites and global serum metabolites, which resulted in a set of 75 correlated serum metabolites (Fig. 3A and Supplementary Table S4). Finally, to ascertain that the identified serum metabolites can stratify patients into similar subgroups as those defined by metabolic genes and tumor tissue metabolites, we performed consensus clustering analysis based on the 75 serum metabolites, which similarly yielded three stable clusters of the samples named SerumMet-C1, SerumMet-C2, and SerumMet-C3. Similar to our assessment of metabolic genes, we found a concordance between good and poor survival subclasses defined by the 75 serum metabolites and those defined by published signatures.

We also investigated the mutation status of two genes that often mutated in HCC, namely TP53 and CTNNB1. These two genes are not usually mutated together in the same patient. We found that TP53 mutation are predominantly in MetGene-C1, TissueMet-C1, and SerumMet-C1 clusters, whereas CTNNB1 mutation are predominantly found in MetGene-C3, TissueMet-C3, and SerumMet-C3 clusters (Fig. 2B—lower panel).

**Figure 4:**
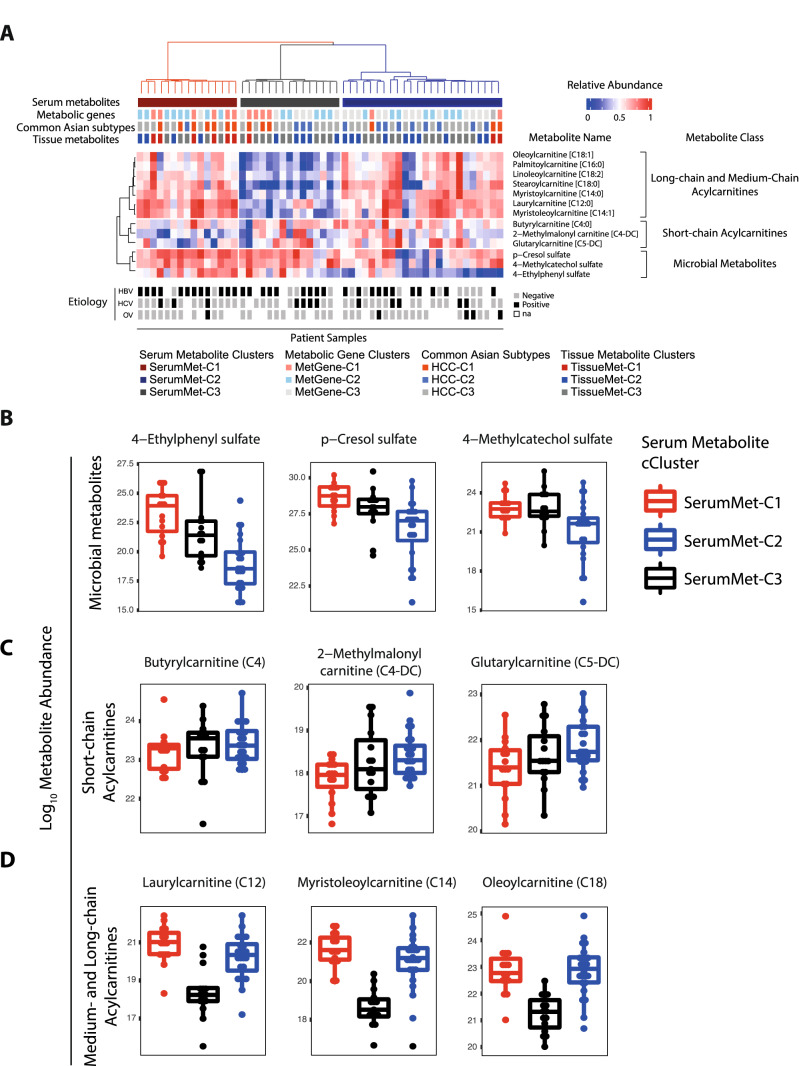
Patient clustering by serum metabolites and identification of prognostic serum metabolites. (**A**) Heatmap of metabolite abundance from three selected groups of metabolites of interest: short-chain acylcarnitines; long-chain acylcarnitines; and microbial metabolites. The top panel is patient clusters based on various consensus clustering results: first row is consensus clustering results based on 75 serum metabolites; second row is consensus clustering results based on 491 metabolic genes; third row is the original consensus clustering of TIGER-LC HCC samples; and fourth row is consensus clustering results based on 40 tumor-tissue metabolites. (**B**–**D**) Log-scale metabolite abundance. (**B**) Microbial metabolites (p-cresol sulfate, 4-ethylphenyl sulfate, and 4-methylcatechol sulfate). (**C**) Short-chain acylcarnitines (Butyrylcarnitine [C4:1], 2-methylmalonyl carnitine [C4-DC] and Glutarylcarnitine [C5-DC]). (**D**) Medium- (laurylcanitine [C12:1]) and long-chain acylcarnitines (Myristoleoylcarnitine, [C14:1] and Oleoylcarnitine [C18:0]. The y-axis is the log10 metabolite abundance. The x-axis is the SerumMet clusters. Abbreviations: MetGene-C—Metabolic gene-Cluster; TissueMet-C—Tissue metabolite-Cluster; SerumMet-C—Serum metabolite-Cluster; HCC—Hepatocellular Carcinoma.

To determine whether the resulting patient subgroups are associated with patient outcome, survival analysis was performed. Survival analysis based on consensus clusters revealed that the C1 cluster from metabolic genes (MetGene-C1), tissue metabolites (TissueMet-C1), and serum metabolites (SerumMet-C1) have similarly worse survival outcome compared to other clusters (Fig. 3B–D, respectively). Hazard ratios [with 95% confidence interval (CI)] between the C1 cluster and other clusters defined by metabolic genes, tumor-specific tissue metabolites, and serum metabolites are 5.8 [2.2–15], 3.3 [1–10], and 4.4 [1.6–12] respectively. The same trends were also observed in the LCI and TCGA-Asian cohorts, however the C3 cluster was not present in either cohort (Supplementary Fig. S3E, F). The C2 and C3 clusters from both metabolic genes and tissue metabolites of the TIGER-LC cohort have similar survival trajectories (MetGene-C2 and MetGene-C3 in Fig. 3B, and TissueMet-C2 and TissueMet-C3 in Fig. 3C), which should be expected since these metabolic genes and metabolites are correlated. The trajectory of the C3 cluster in serum metabolites, however, did not resemble those of metabolic genes or tissue metabolites clusters (Fig. 3D). Traditional prognostic biomarker for HCC, i.e., AFP > 300 ng/ml, cannot separate patients into two subgroups with statistical significance in this cohort (Supplementary Fig. S4). Hazard Ratio [95%CI] for AFP as prognostic biomarker in this cohort is 2 [0.72–5.5]. Interestingly, the samples that were classified as SerumMet-C1 using serum metabolites were predominantly C1 samples identified by metabolic genes, tissue metabolites, and common Asian subtypes in our previous study^13^, and the samples from the C1 cluster classified by tissue metabolites are consistent with the C1 samples from serum metabolites (Fig. 4A—upper panel). Similar to the consensus clusters identified using metabolic genes, there is no discernible pattern associated with any cluster defined by the serum metabolites and standard HCC etiology parameters (Fig. 4A—lower panel). Overall, serum metabolites were representative of metabolic genes and tissue metabolites in their capacity to classify outcome-related molecular subgroups.

### Identified serum metabolites are associated with fatty acid metabolism and secondary metabolism of microbial metabolites

We next ascertained the pathways that were present and/or altered among the metabolite-defined clusters. To do so, we performed pathway analysis based on the metabolic gene expression among the MetGene clusters. When comparing the 491 metabolic gene expression between samples from different MetGene clusters using Ingenuity Pathway Analysis (IPA)^25^, we found that the top three pathways, namely lipid metabolism, i.e. LPS/IL-1 mediated inhibition of RXR function, fatty acid β-oxidation I, and PXR/RXR activation, were deregulated in all the comparisons between the three MetGene clusters, with − log_10_(*p*-values) of MetGene-C1/C2, MetGene-C1/C3, and MetGene-C2/C3 as 21.7, 24.4, and 21.7, respectively (Supplementary Fig. S5 and Supplementary Table S5). The results concurred with serum metabolite data, where short-chain and long-chain acylcarnitines were differentially abundant among the three serum metabolite clusters (Fig. 4A and Supplementary Fig. S6).

To further confirm that these dysregulated pathways based on gene expression levels are truly associated with lipid metabolism, we determined the composition of the identified 75 serum metabolites by tabulating the metabolites according to their classes and subclasses. Almost half (46%) of the identified serum metabolites were lipids (Supplementary Fig. S2B), which consisting mainly of acylcarnitines (28%), phospatidylcholine and lysophosphatidylcholine (PC and lysoPC—25%), while the remainder are other assorted lysolipids, steroids and fatty acids (Supplementary Fig. S2C). One class of serum metabolites with highest abundance, namely acylcarnitines, is strongly correlated with tissue metabolites (Fig. 4C, D) and reflect the pathway analysis based on tumor and adjacent non-tumor matched tissue gene expression, with HNF4α as possible transcriptional regulator (Supplementary Figs. S7A, B and S8). Notable metabolites from this class are short-chain acylcarnitines (butyrylcarnitine, 2-methylmalonyl carnitine, and glutarylcarnitine) and long-chain acylcarnitines (laurylcarnitine, myristoleoylcarnitine, and oleoylcarnitine). The median relative abundance levels in log10 of these metabolites for SerumMet-C1, C2, and C3 are as follow: butyrylcarnitine—23.32, 23.44, 23.50; 2-methylmalonyl carnitine—17.9, 18.25, 18.13; glutarylcarnitine—21.40, 21.81, 21.59; laurylcarnitine—21.0, 20.42, 18.23; Myristoleoylcarnitine—21.81, 21.67, 18.23; and Oleoylcarnitine—22.87, 22.92, 21.35. Acylcarnitines are intermediate metabolites in fatty acid metabolism, specifically in process of fatty acid transportation from the cytosol to the mitochondria during fatty acid β-oxidation whereby fatty acids are broken down. The next most abundant class of serum metabolites is lysoPC, which constitutes the majority of biological membranes and is easily obtained through the diet. Other metabolite classes were amino acids, xenobiotics, peptides, carbohydrates, cofactors and vitamins, and energy metabolites. One other notable class of metabolites observed among the 75-serum metabolite set is microbial metabolites, which consisted of *p*-cresol sulfate (PCS), 4-ethylphenyl sulfate (4-EPS), and 4-methylcathechol (Fig. 4A, B, Supplementary Fig. S9 and Supplementary Table S5). The median relative abundance levels in log10 of these metabolites for SerumMet-C1, C2, and C3 are as follow: p-cresol sulfate—28.62, 26.95, 27.72; 4-ethylphenyl sulfate—23.84, 18.32, 21.05; and 4-mythylcatechol sulfate—22.64, 21.72, 22.51.

Finally, to determine whether clinical variables were associated with various subtypes identified in this study, we performed a Fisher’s Exact test between these clinical variables and the subtypes. We found that most of the clinical features are independent of these subtypes, with the two exceptions of AFP/MetGene subtypes and BMI/SerumMet subtypes (Supplementary Table S5). The samples with AFP level > 300 ng/ml are clustered in the MetGene-C1 subgroup, which has worse prognosis compared to other subgroups. The group with BMI > 24 (overweight patients) is linked with the SerumMet-C2 subgroup, which has better prognosis compared to other subgroups. This data concur with the notion that short-chain acylcarnitines in SerumMet-C2 subgroup have the most abundant level among all the subgroups.

## Discussion

In this study, we present a set of serum metabolites that can potentially be used as non-invasive prognostic biomarkers that are reflective of tumor-tissue status. The multiple levels of metabolite-related data can independently stratify Asian HCC patients into molecular subtypes that have similar patient outcomes. Compared to our previous study that utilized whole transcriptome analysis to identify HCC molecular subtypes^13^, here, we have considerably decreased the number of markers that are required for patient stratification without compromising the subgroup consistency. Furthermore, the identified metabolic genes and metabolites gave new insights into the molecular underpinnings of Asian HCC.

Serum metabolite measurement is a potential non-invasive tool for early detection and prognosis determination of HCC. Compared to other noninvasive techniques, such as ultrasonography, computed tomography, or magnetic resonance imaging (MRI), serum metabolites are considerably less cumbersome in terms of implementation and results interpretation. Previous publications have identified several fatty acids and acylcarnitines in serum and plasma as early detection and prognostic biomarkers of HCC, indicating that fatty acid metabolism is universally dysregulated in HCC^12,26–29^. However, to our knowledge, there is no study which directly correlates the chain of events between gene expression and metabolites from tumor tissues to serum. Our study therefore may present a more accurate picture of the molecular underpinnings of HCC.

The methodology employed in this study, namely two-step correlation analysis by first correlating tissue gene expression and metabolite and then correlating tissue metabolites and serum metabolites, has unique advantages over single step correlation. This approach combines permutation-based correlation threshold selection with correlation analysis to objectively identify correlated genes and metabolites. The approach also utilized tissue gene expression, tumor tissue and serum metabolites to identify the plausible causal chain of events that should directly reflect molecular processes from tumor tissues to tissue metabolites, and finally to serum metabolites. To our knowledge, this is the first study that utilized these data in this way. This method, combined with gene expression and metabolomics data, allows us to directly observe the chain of associations between gene expression and metabolites in tumor tissue, and between tumor tissue and serum metabolites. It enables us to see a convergence of evidence from multiple levels of data, from which patient subgroups resulting independently from metabolic gene expression, tissue, and serum metabolites had similar patient outcome.

The set of 75 serum metabolites was able to independently stratify patients into subgroups that were also consistent with patient subgroups from metabolic gene expression, tumor tissue metabolites, and whole transcriptome from our previous study^13^. The consistency in these clustering results is another indicator that the identified metabolic genes and metabolites are involved in the disease manifestation. Furthermore, the numbers of genes and metabolites involved in sample stratification were significantly lower compared to our previous study, which will be beneficial for further utilization of these genes and metabolites as prognostic biomarkers. Finally, serum metabolites are more accessible and less invasive than gene expression or metabolites from tumor tissues, therefore they are more suitable as less invasive prognostic biomarkers for Asian HCC patients. This set of serum metabolites can be validated in larger patient cohorts in the future to facilitate HCC patient prognostication in clinical settings.

Lipids and fatty acid metabolism play central roles in all cancer types, including HCC^30^. Pathway analyses based on the abundance of both tumor tissue and serum metabolites, combined with tissue gene expression, indicated that lipid and fatty acid metabolism are among the top dysregulated metabolic pathways in HCC. The roles of one class of fatty acid-related metabolites of interest, i.e., acylcarnitines, are slowly emerging in HCC. Acylcarnitines are known as intermediate metabolites in β-oxidation of fatty acids in mitochondria^31,32^. Short- and medium-chain acylcarnitines largely reside in peroxisomes and produced mainly by liver and muscle^33,34^. A recent study showed that acylcarnitine profiles can be used as biomarkers for obesity-driven HCC, pinpointed a serum metabolite, oleoylcarnitine (C18:1 carnitine), which was found in this study, as one of the biomarkers for NAFLD and HCC, and revealed the mechanism of actions of oleoylcarnitine through STAT3 pathway activation^31^. The same study also identified several other acylcarnitines that were also identified in this study, namely laurylcarnitine (C12:0) and myristoleoylcarnitine (C14:1), as possible HCC biomarkers. For short- and medium-chain acylcarnitines, the evidence of the involvement in HCC are limited. Possible master regulators for these metabolites are HNF4α, which were found in the pathway analysis and interacts with CPT1 and CPT2. HNF4α was found to be the main regulator for acylcarnitines in brown adipose tissue in mice, in which the brown tissue increases the uptake of acylcarnitines during the cold^35^. This will need further analyses to confirm the actual link between HNF4α and acylcarnitines in human HCC.

The gut microbiome has been shown to play several important roles in liver diseases. Several mechanisms involving the gut microbiome have been proposed, along with approaches to mitigate, prevent, or even reverse HCC^36^. Many essential metabolites and nutrients are produced by gut microbes to provide precursors for more complex molecules in human, such as hormones, vitamins, and other cofactors. Some metabolites identified in this study, i.e., 4-EPS and PCS, are microbial metabolites derived from the diet^37^. Several studies found that PCS is a highly potent uremic toxin^38^ and can induce cell proliferation and migration of kidney cancer^39^. The roles of these compounds in HCC are still obscure. *p*-cresol is produced mainly by *Clostridioides difficile* (also known as *Clostridium difficile* or *C. diff*)^40^. A recent study predicted that there may be other microbes which can also produce *p*-cresol, but this needs to be confirmed^41^. *p*-cresol is metabolized in the liver through a conjugation process, i.e. sulfonation, to produce a less toxic form, PCS^42^. There are several possible mechanisms that might be specifically linked to PCS. It was found through molecular docking simulations that PCS can activate epidermal growth factor receptor (EGFR) via interdomain pocket interaction of extracellular EGFR, and finally induce kidney tissue remodeling^43^. A recent study showed that EGFR signaling promotes survival of HCC cell lines via interaction with E-cadherin and β-catenin, but only in late stages of the disease^44^. Another possible mechanism is that PCS might increase liver detoxification burden, especially sulfonation, affecting the ability of liver to detoxify other highly potent liver toxins, such as acetaminophen (paracetamol) or aflatoxin^45^. This study also proposed a less invasive means for measuring PCS through urine in HCC patients^45^. PCS may also be associated with the microbiome in that PCS-producing *C. diff* was found to be dominating in the gut of mice, due to toxicity of PCS to other bacterial species in the gut^46^ and may be the mechanism of action of dysbiosis from *C. diff* infection. Therefore, PCS might be a key link between gut dysbiosis and HCC pathogenesis.

Biomarker discovery for HCC is an active field of research. The metabolites mapped onto the KEGG’s Human Metabolism Map in Fig. 1C were previously found to be dysregulated in HCC and have been proposed as either prognostic or diagnostic biomarkers, but independently they were not able to outperform traditional HCC biomarkers. To further explore these dysregulations, we have assessed tumor specific metabolic gene alterations and serum alterations and determined their association with clinical features including patient outcome. The specific alterations and how they apply to HCC tumorigenesis would require detailed molecular and functional studies which could be addressed in future studies. Numerous studies have been conducted to search for suitable serum biomarkers that can predict survival outcome of patients in different populations^47–50^. Currently, however, clinically accepted prognostic serum biomarkers are limited to only α-fetoprotein (AFP), AFP-L3, and Des-γ-carboxy prothrombin (DCP)^51,52^. Various acylcarnitines were suggested as serum or plasma biomarkers in several Asian HCC cohorts, but none of these biomarkers have been validated in any clinical trials^29,53,54^. The main reason for this may result from the inconsistency of the data from these studies. One study of PCS suggests that it could be a urine-based biomarker for differentiating HCC and cirrhotic patients. However, the study found that HCC patients have lower PCS level compared to cirrhotic patients and suggested that sulfonation alteration might be responsible mechanisms^55^. Thus, additional comprehensive studies are needed to determine the consistency and effectiveness of such biomarkers.

In conclusion, we have established stable metabolic subtypes based on metabolic gene expression, tissue metabolites and importantly, serum metabolites, through a two-step correlation analysis. Metabolic gene subtypes can stratify patients into better and worse prognosis based on the metabolic gene expression across different Asian patient cohorts. Tissue and serum metabolites can also consistently stratify patients into subgroups similar to the metabolic gene subtypes and our previously identified common molecular Asian transcriptome-defined subtypes. Some of the serum metabolites that we identified might serve as prognostic biomarkers after future clinical validation. Validation in other cohorts should be a priority to translate this finding into clinical use. Publicly available resource with both gene expression and serum metabolite measurement are not currently available to further validate our findings. If and when such resources are available, we should have clearer picture of the serum metabolites associated with HCC. Finally, future studies that link metabolic alterations with disease etiologies and clinical features would be of future interest to shed light on the underlying mechanisms contributing to HCC which could be utilized in clinical management.

## Methods

### Cohorts, clinical, and genomic data

Tissue gene expression of the TIGER-LC cohort^13^, LCI cohort^20^ and TCGA liver cohort^24^, were used this analysis. The list of metabolic genes (3765 genes) were retrieved from Human Metabolic Reaction 2.0 (HMR2.0) database^56^. Tissue metabolite data were described previously among the TIGER-LC cohort^13^. In brief, metabolites were measured in tumor tissues and serum using Metabolon’s Discover HD4 Platform, which is an untargeted metabolomics approach that combines positive and negative modes of liquid chromatography (LC+/LC−) and gas chromatography/mass spectrometry (GS/MS) to measure metabolite abundance. After data processing, 718 and 990 metabolites were detected from tumor tissues and serum samples, respectively. After excluding unidentified metabolites (metabolites that cannot be matched to standard library compounds), there were 562 and 615 metabolites remaining for further analyses from tumor tissues and serum, respectively. Clinical features data included in this study are

1. Mutation status of TP53 and CTNNB1 genes, determined in our previous study^13^
2. Sex
3. Age, with two categories of <  = 50 and > 50 years old
4. Body Mass Index (BMI), with two categories of BMI <  = 24 and BMI > 24
5. Diabetes status, with two categories of no diabetes and with diabetes
6. CA-19–9 level, with two categories of CA-19–9 <  = 37 U/ml and CA19-9 > 37 U/ml
7. AFP level, with two categories of AFP <  = 300 ng/ml and AFP > 300 ng/ml
8. Cirrhosis status, with two categories of Cirrhosis Positive and Cirrhosis Negative
9. TNM classification of malignant tumors, with two categories of early stage I and II, and late stage of IIIA, IIIB, and IV
10. Hepatitis B virus (HBV) infection status, with two categories of HBV Positive and HBV Negative
11. Hepatitis C virus (HCV) infection status, with two categories of HCV Positive and HCV Negative
12. *Orpisthorchis viverrini* (OV) or liver fluke infection status, with two categories of OV Positive and OV Negative

### 3D PCA of tissue metabolites

Three-dimensional principal component analysis projections of tumor and adjacent non-tumor tissue metabolites were constructed based on the 500 most variable metabolites. The most variable metabolites were selected based on variance of each metabolites among all HCC samples.

### KEGG human and microbial metabolic maps

The KEGG map overlay with metabolite ratio was performed using the R package pathview^57^. The metabolite names are converted to KEGG compound IDs, and the maps required are downloaded through KEGG API by KEGGREST package in R^58^. The tissue metabolites from the TIGER-LC cohort were overlaid onto the KEGG human metabolite map^15^ (map accession number: hsa01100, retrieved on April 5, 2017) and reconstructed based on the TIGER-LC tissue metabolite ratio between the mean of tumor tissues and adjacent non-tumor tissues. The log2-ratio of metabolites between tumor and adjacent non-tumor tissues were calculated and used for map overlay. The serum metabolites were overlaid onto the KEGG Microbial Metabolism in Diverse Environments map (map accession number: KO01120, retrieved on April 10, 2021) and reconstructed based on the serum metabolite ratio between samples from SerumMet-C1 and SerumMet-C2 clusters.

### Two-step correlation analysis and correlation coefficient threshold selection by permutation analysis between metabolic gene expression, tissue metabolites, and serum metabolites

There are 3765 genes classified as human metabolic genes according to Human Metabolic Atlas database version 2.0^59^, of which approximately 2000 genes overlapped with the gene expression data from our previous study^13^. Metabolic gene expression between tumor and adjacent non-tumor tissue were compared using a paired t-test. The resulting *p*-values were adjusted using Benjamini–Hochberg false discovery rate (FDR) procedure^60^. Metabolic genes with FDR-adjusted *p*-values of less than 0.05 were retained for further correlation analysis. Spearman correlation coefficients between the expression of 2300 metabolic genes from tumor tissues and 562 metabolites from the TIGER-LC cohort were calculated.

For correlation threshold selection, the gene names, metabolite names, and the sample names in both metabolic genes and metabolite matrices were permutated to destroy original relationships of the original data. Then, the correlation coefficient between permutated metabolic gene expression and metabolites were calculated and recorded with 10,000 permutations. The distribution of correlation coefficients from the original data was plotted against the average of distributions of correlation coefficients from the permutated data. Finally, the intersection points of correlation coefficients between the original data and the average of permutated data was determined to be -0.165 and 0.165 (Supplementary Fig. S10) and were used as correlation coefficient thresholds to select the correlation coefficients between metabolic genes and metabolites for further analyses.

The metabolic genes and tissue metabolites were selected by calculating the average value of the sum of absolute correlation coefficients from the following equations:

$$G_{i} = \frac{{\mathop \sum \nolimits_{j = 1}^{J} \left| {c_{ij} } \right|}}{J}\;\;{\text{and}}\;T_{j} = \frac{{\mathop \sum \nolimits_{i = 1}^{I} \left| {c_{ji} } \right|}}{I}$$

where |*c*_*ij*_| and |*c*_*ji*_| are absolute correlation coefficient between metabolic gene *i* and tissue metabolite *j*, *I* is total number of metabolic genes, *J* is total number of tissue metabolites, *G*_*i*_ is the average value of sum of absolute correlation coefficients of metabolic gene *i* over tissue metabolites, and *T*_*j*_ is the average value of sum of absolute correlation coefficients of tissue metabolite *j* over all metabolic genes. *G* and *T* that are higher than the correlation coefficient threshold identified by permutation test are then selected for further analyses.

For the first step of correlation, the number of metabolic genes and tissue metabolites from correlation coefficient threshold selection is 491 genes and 78 metabolites, respectively. Tissue metabolites that are tumor-specific was determined based on a metabolite fold-change of 1.5 between tumor and non-tumor adjacent tissues, which yielded 40 tumor-specific tissue metabolites that were used in the second step of correlation with serum metabolites.

For the second step of correlation, correlation thresholds used for correlation analysis between tissue and serum metabolites are − 0.15 and 0.15. These thresholds correspond to the sums of correlation coefficients between tumor tissue gene expression and tissue metabolites previously identified. Serum metabolite selection, the same strategy of average of the sum of absolute correlation coefficient is used, following the equation:

$$S_{k} = \frac{{\mathop \sum \nolimits_{m = 1}^{M} \left| {c_{km} } \right|}}{M}$$

where |*c*_*km*_| is absolute value of correlation coefficient between serum metabolite *k* and tumor-specific tissue metabolite *m*, *M* is total number of tumor-specific tissue metabolites, and *S*_*k*_ is average value of sum of absolute correlation coefficient of serum metabolite *k* over all tumor-specific tissue metabolites. The final number of serum metabolites that passed the threshold is 75 metabolites.

### Consensus clustering analysis and subclass mapping analysis

To define consensus clusters for the patient samples among the Thai cohort, the matrix of 491 metabolic genes was used as the input for consensus clustering^61^. Two other cohorts were used to validate identified metabolic genes, namely TCGA-LIHC^24^ and LCI^20^ cohorts. The TCGA-LIHC cohort is comprised of liver cancer patients from across US cancer centers as a part of The Cancer Genome Atlas project. The LCI cohort is comprised of Chinese liver cancer patients from the Liver Cancer Institute. In our previous study we found that Asian patients had common molecular features, which are different than patients with European or African descent^13^. Therefore, in this study we only used Asian descent samples from TCGA-LIHC. The data matrices from these two cohorts were obtained by using the original 491 genes from the TIGER-LC cohort analysis and identifying the overlapping genes from the TCGA-LIHC or LCI data sets. The LCI cohort has 385 overlapping genes, while TCGA-LIHC cohort has 461 overlapping genes. Consensus clustering was performed by using ConsensusClusterPlus^16^ package version 1.38 in R version 3.3.3. The reason for using consensus clustering in this study is to determine whether the sets of metabolic gene expression, tumor tissue, and serum metabolites can independently stratify patients into subgroups that have similar consistency amongst different molecular data types. The parameters used in all the analyses were as follows: maxK = 8, number of bootstrap = 20,000; item subsampling proportion = 0.8; feature subsampling proportion = 1; cluster algorithm = hc [hierarchical clustering]; inner linkage type = ward.D; final linkage type = ward.D; correlation method = spearman; seed = 3044. The final number of clusters (k) selected for further analyses is three.

The clustering results from the LCI and TCGA-Asian cohorts were then subjected to Subclass Mapping (SubMap)^62^ analysis to determine whether the clusters from these two cohorts matched the clusters from the TIGER-LC cohort. SubMap version 7.0 from GenePattern software suite^63^ was used with default parameters. After two-step correlation analysis, the list of 40 tumor-specific tissue metabolites and 75 serum metabolites were also used for consensus clustering with the same parameters previously described to confirm that the metabolites identified by correlation analysis can independently and consistently stratify patients into similar subgroups. Submap analysis was also used to determine the if the TIGER-LC samples matched with other HCC signatures. The HCC signatures used in this study were S1-S3 signature based on WNT-TGFβ, AKT-MYC signaling pathway activation, and hepatocyte differentiation^17^, stem cell signature based on expression of stem cell gene set^18^, EpCAM signature based on a gene set that is co-expressed with EpCAM^19^, metastatic signature based on a gene set related to metastasis of HCC^20^, CCA-like signature based on a gene set from cholangiocarcinoma-like HCC^21^, Cluster 1 and 2 signatures based on 238-gene signature from CCA patients^22^, and proliferation signature based on copy number alterations and activation of oncogenic pathways^23^.

### Statistical and survival analyses

All statistical and survival analyses were performed using R statistical computing software version 3.3.3. Survival analysis on all cohorts was performed using a Kaplan Meier estimator and a Cox proportional hazards model was calculated between the worst survival group and the rest of the groups, accompanied with Log-rank *p*-value for each comparison. The Cox proportional hazard assumption was tested and met for all the tests carried out. The Paired t-test was used to calculate differential gene expression between tumor and adjacent non-tumor tissues. All *p*-values are two-sided *p*-values unless otherwise stated and adjusted for false discovery rate by Benjamini–Hochberg FDR procedure^64^.

### Pathway analyses

The final 491 metabolic genes were subjected to pathway analyses using GSEA^65^ version 18 on GenePattern software suite^63^ for tumor vs non tumor comparison, and Ingenuity Pathway Analysis (IPA)^66^ version 01-16 for tumor clusters comparison.

## Supplementary Information

Supplementary Information 1.

Supplementary Information 2.
