# Supplementary material for: Tumor metabolism and associated serum metabolites define prognostic subtypes of Asian hepatocellular carcinoma
Source: Sci Rep. 2021 Jun 8;11:12097. doi: 10.1038/s41598-021-91560-1 (PMC8187378; doi:10.1038/s41598-021-91560-1)

Tumor Metabolism and Associated Serum Metabolites Define Prognostic Subtypes of Asian Hepatocellular Carcinoma

Supplementary Figures S1-S10

Yotsawat Pomyen[1,2,&], Anuradha Budhu[1,3,&], Jittiporn Chaisaingmongkol[1,4,5], Marshonna Forgues[1], Hien Dang[1,+], Mathuros Ruchirawat[4,5], Chulabhorn Mahidol[4], and Xin Wei Wang[1,3,*]
on behalf of the TIGER-LC Consortium[#]

[1]Laboratory of Human Carcinogenesis, Center for Cancer Research, National Cancer Institute, Bethesda, MD 20892, USA

[2]Translational Research Unit, Chulabhorn Research Institute, Bangkok, 10210, Thailand

[3]Liver Cancer Program, Center for Cancer Research, National Cancer Institute, Bethesda, MD 20892, USA

[4]Laboratory of Chemical Carcinogenesis, Chulabhorn Research Institute, Bangkok 10210, Thailand

[5]Center of Excellence on Environmental Health and Toxicology, Office of Higher Education Commission, Ministry of Higher Education, Science, Research and Innovation, Bangkok 10400, Thailand

[&]These authors contributed to the work equally

[+]Present address: Division of Surgery, Thomas Jefferson University, Philadelphia, PA, 19107, USA

*Correspondence: xw3u@nih.gov

[#]Lead contacts of TIGER-LC consortium: XWW (xw3u@nih.gov) and MR (mathuros@cri.or.th)

Supplementary Figure Titles and Legends

Supplementary Figure S1 – Detailed study scheme. [related to Figure 1A]

Detailed overall flowchart of the analysis, where rectangles represent processes in the analysis, parallelograms represent inputs into a process, rectangles with wavy base represent list of genes or metabolites that resulting from a process. The numbers on top right of the parallelograms represent number of genes or metabolites as the inputs to a process. The colors of the input parallelograms represent different cohorts; red represents TIGER-LC, blue represents LCI, and green represents TCGA-LIHC. The left panel of the dash line used data from tissue samples, whereas the right panel of the dash line used data from serum samples

Supplementary Figure S2 – Tissue and serum metabolite classes and subclasses. [related to Figure 2A]

The classes of (A) tumor-specific-tissue metabolites and (B) serum metabolite that were identified by correlation analysis, with (C) lipid subclasses in the serum metabolites. PC – Phosphatidylcholine; PE – Phosphatidylethanolamine; PG – Phosphatidylglycerol; PI – Phosphatidylinositol.

Supplementary Figure S3 – Tissue metabolic gene cluster validation cohorts. [related to Figure 2B]

Heatmaps of tumor tissue gene expression from LCI (A) and TCGA-LIHC (Asian patients only) (B) cohorts, with subclass mapping analysis whether the clusters in LCI (C) and TCGA-LIHC (D) cohorts matched the TIGER-LC test cohort, and survival analysis based on the identified clusters of LCI (E) and TCGA-LIHC (F).

Supplementary Figure S4 – KM-plot of AFP.

Kaplan-Meier analysis based on AFP level of the samples. AFP cut-off is set at 300 ng/ml, where blue line represents survival trajectory of patients with AFP ≤ 300 ng/ml and red line represents patients with AFP > 300 ng/ml.

Supplementary Figure S5 – Comparison of altered pathways between metabolic gene clusters. [related to Figures 2B and 4A]

Top ten pathway analysis results by Ingenuity Pathway Analysis (IPA), where each column shows z-scores of the pathways that have been tested between MetGene cluster pairs.

Supplementary Figure S6 – 75-serum metabolite signature. [related to Figure 4A]

Full heatmap of 75 serum metabolites with MetGene, TissueMet, SerumMet clusters and seven HCC signatures on upper panel, and mutation status of TP53 and CTNNB1 genes, clinical features and etiologies on lower panel. Both upper and lower panels used the same information as the main Figure 2C. Each colored box above heatmap represents one sample. The color representing "Good" prognosis and S3 signature is yellow, while "Poor" prognosis and S1 signature is represented by purple. S2 signature is represented by a green box.

Supplementary Figure S7 – Altered lipid metabolism networks in HCC tissues and between metabolic gene clusters. [related to Figures 2B and 4A]

Lipid metabolism gene networks that were identified by IPA based on 491 metabolic genes, comparing tumor and adjacent non-tumor tissues (A) and comparing MetGene-C1/C2 (B). Both

networks have HNF4α gene as a hub for all other genes, which reflects a strong association with acylcarnitines.

Supplementary Figure S8 – Gene set enrichment analysis (GSEA) of metabolic genes in tumor and non-tumor tissues. [related to Figures 2B and 4A]

Pathway analysis results by GSEA, left and right columns are positive enrichment (enriched in tumor tissues) and negative enrichment (enriched in normal tissues), respectively. A) Gene sets based on KEGG database; B) Gene sets based on REACTOME database; C) Gene sets based on Gene Ontology (GO); D) Gene sets based on biological hallmarks.

Supplementary Figure S9 – KEGG Microbial metabolism in diverse environments map

The microbial metabolites from 75 serum metabolites that were able to map onto KEGG's Microbial Metabolism in Diverse Environments (map accession number ko01120[15]).

Supplementary Figure S10 – Permutation of correlation analysis of metabolic genes and tumor tissue metabolites. [related to Figure 1A]

Distribution of correlation coefficients from actual and permutated data. The red line represents the real data distribution, while grey area represents distributions of 10,000 permutation of real data, and black line is an average distribution of the permutation. The two vertical lines are the points that the actual and average permutated distributions crossed, which equate to correlation coefficients of 0.165 and -0.165.

Suppl Fig S1 Detailed study scheme

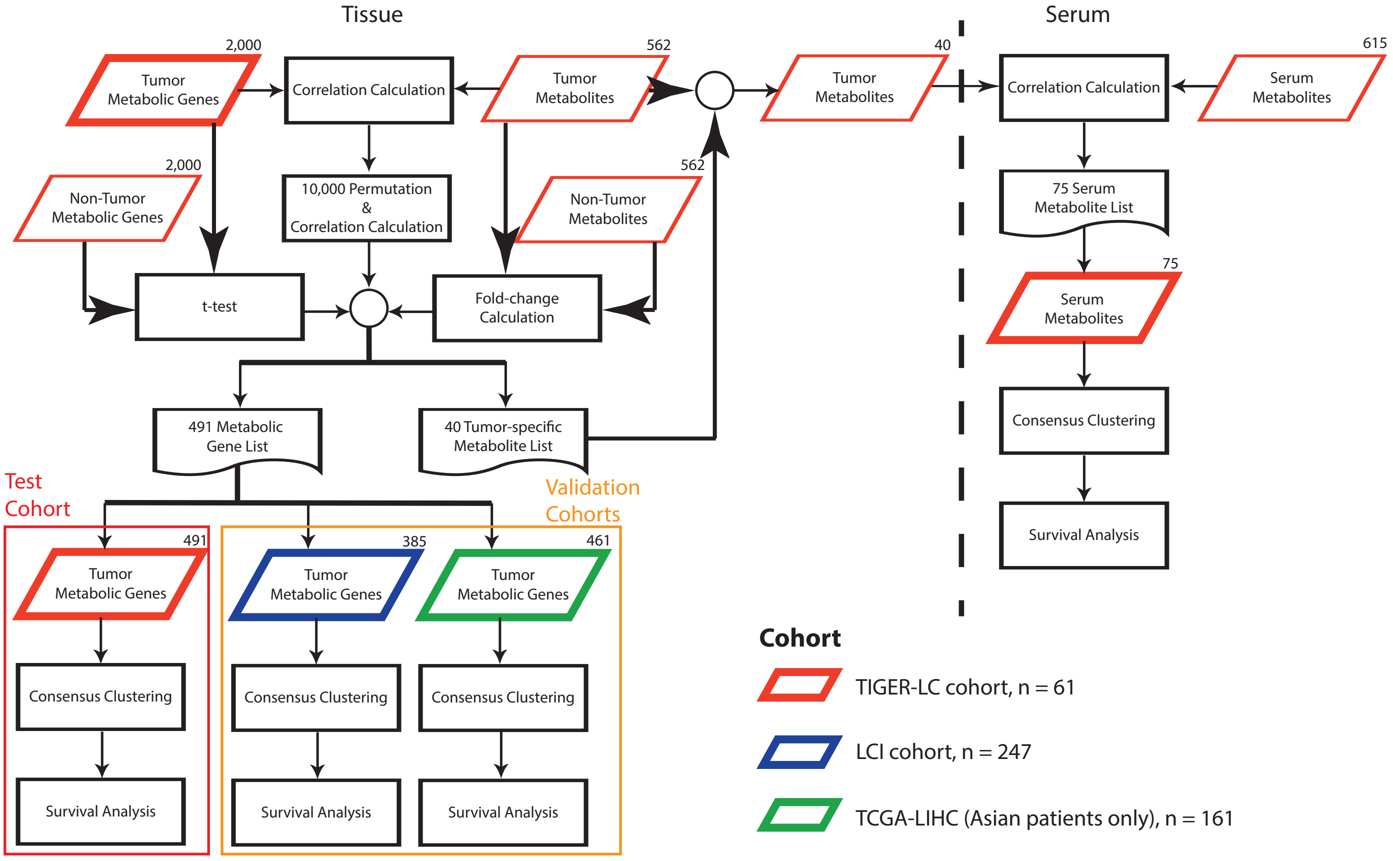

Suppl Fig S2 Tissue and serum metabolite classes and subclasses

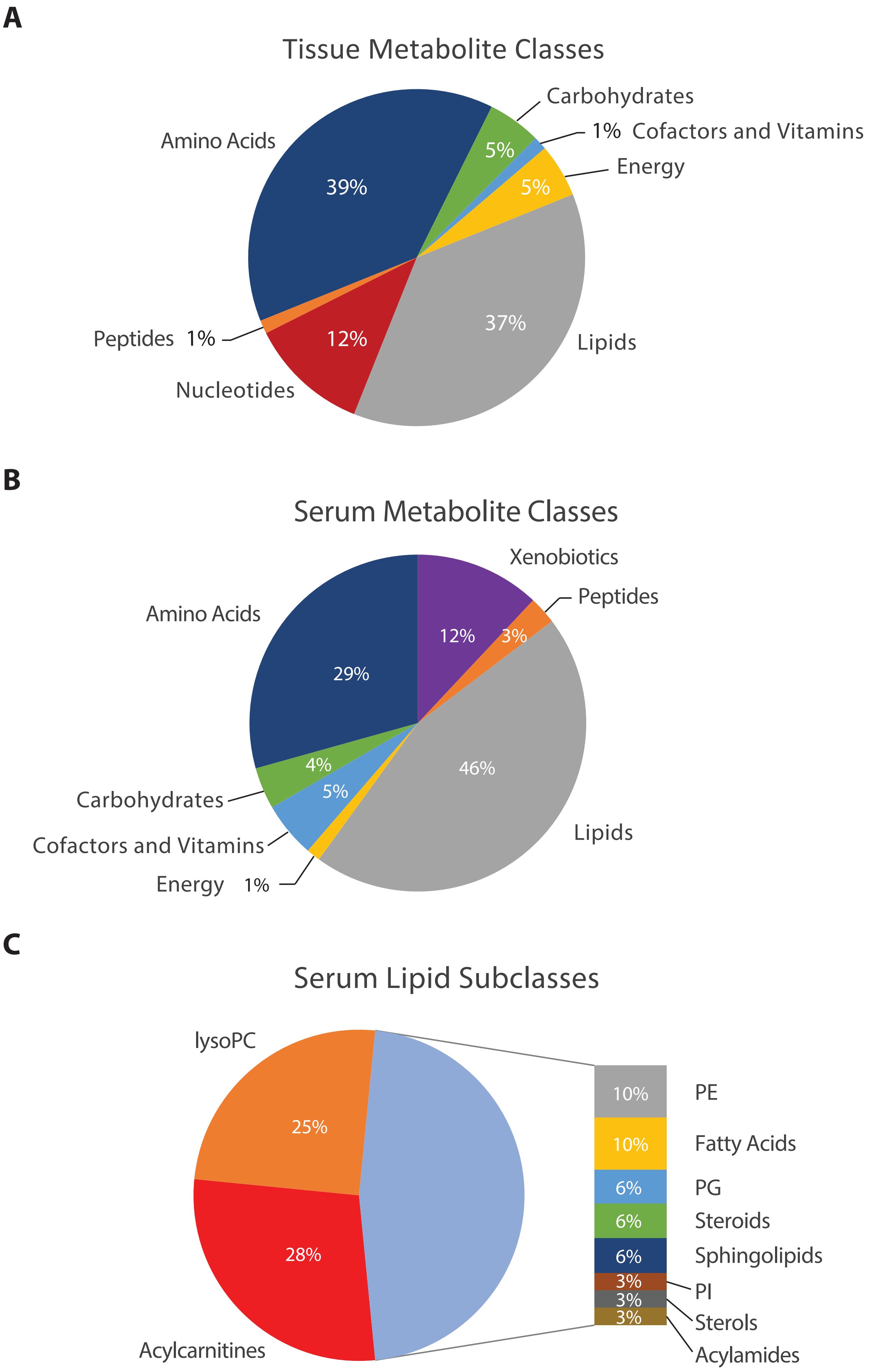

Suppl Fig S3 Tissue metabolic gene cluster validation cohorts

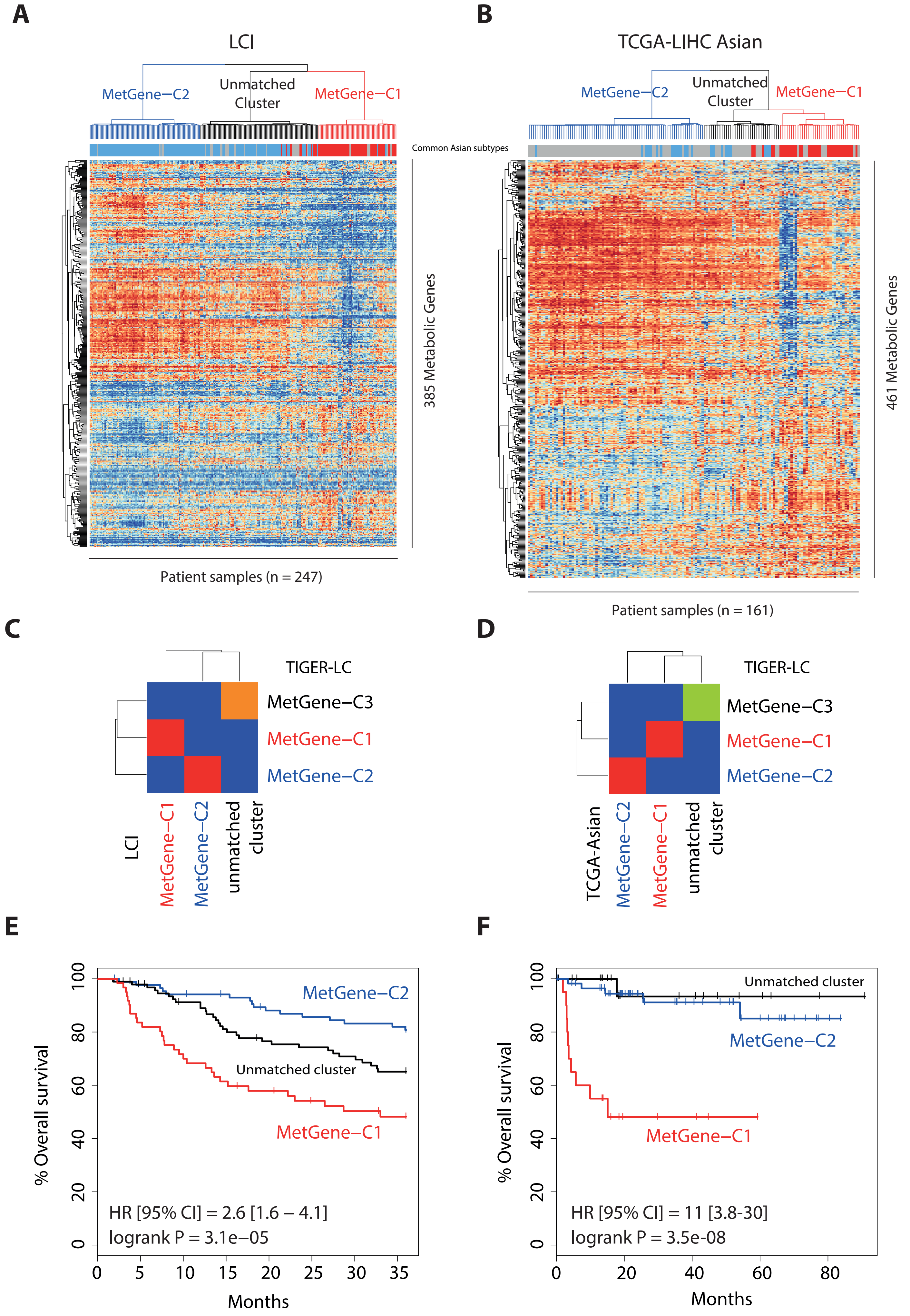

SupplFig S4 KM-plot analysis on AFP

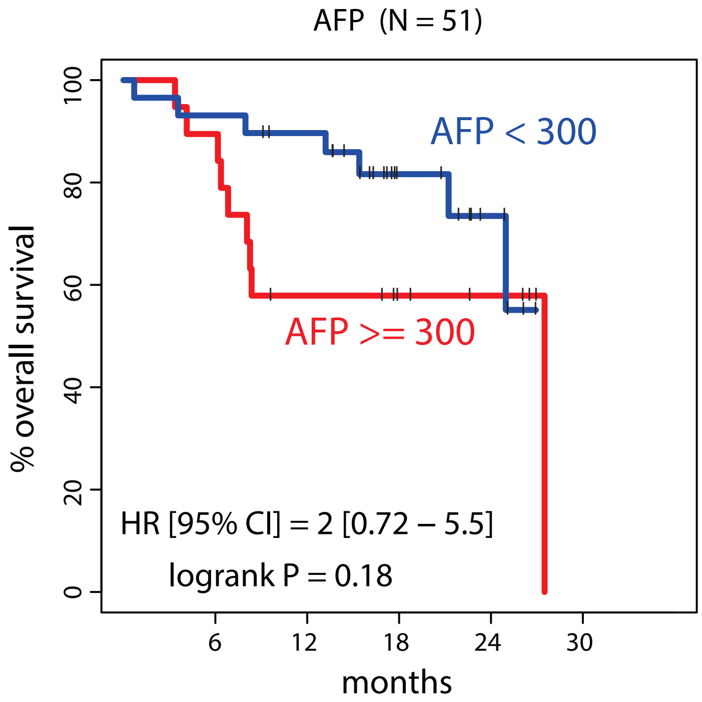

Suppl Fig S5 Comparison of altered pathways between metabolic gene clusters

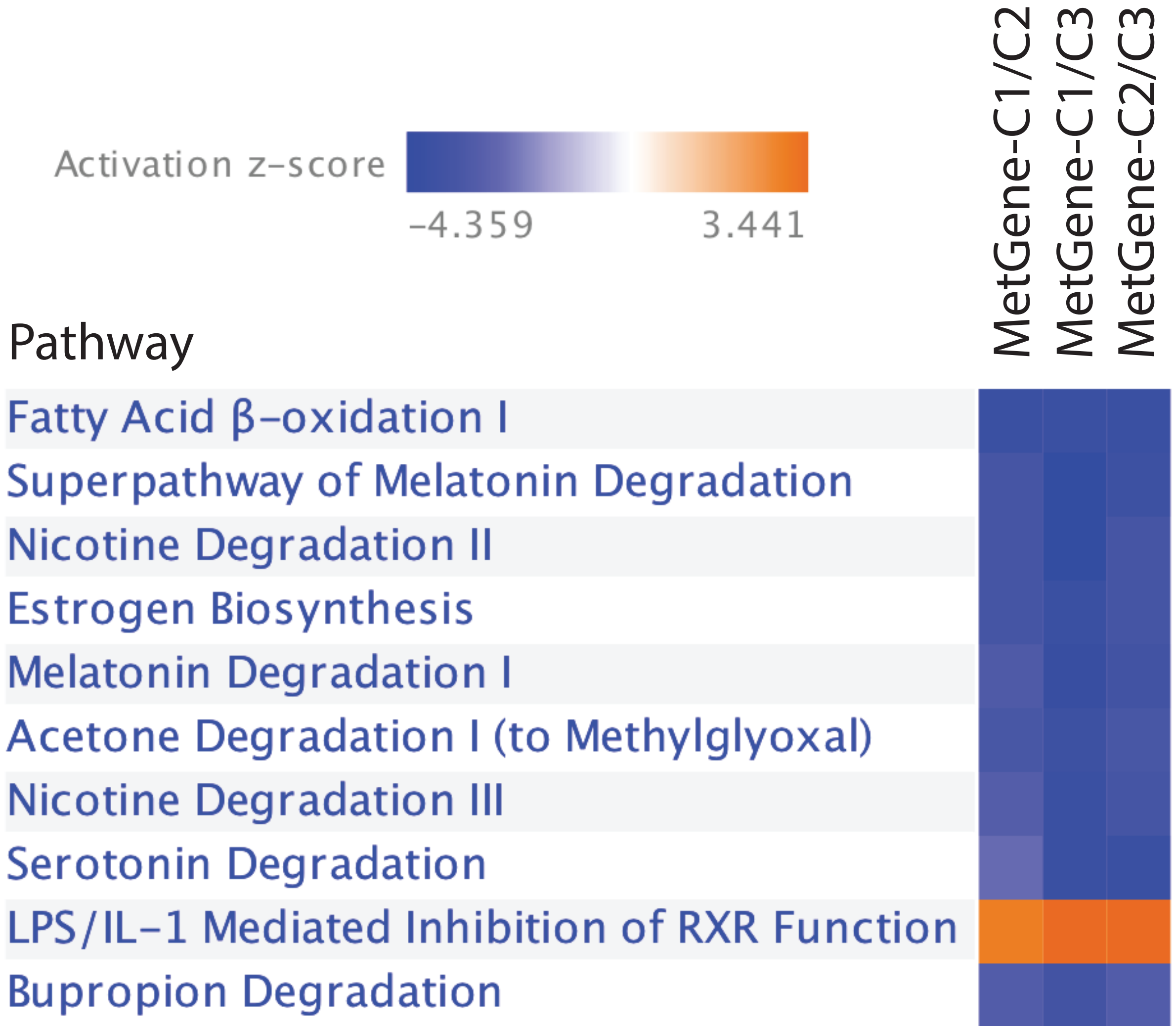

Suppl Fig S6 Heatmap of 75 serum metabolites

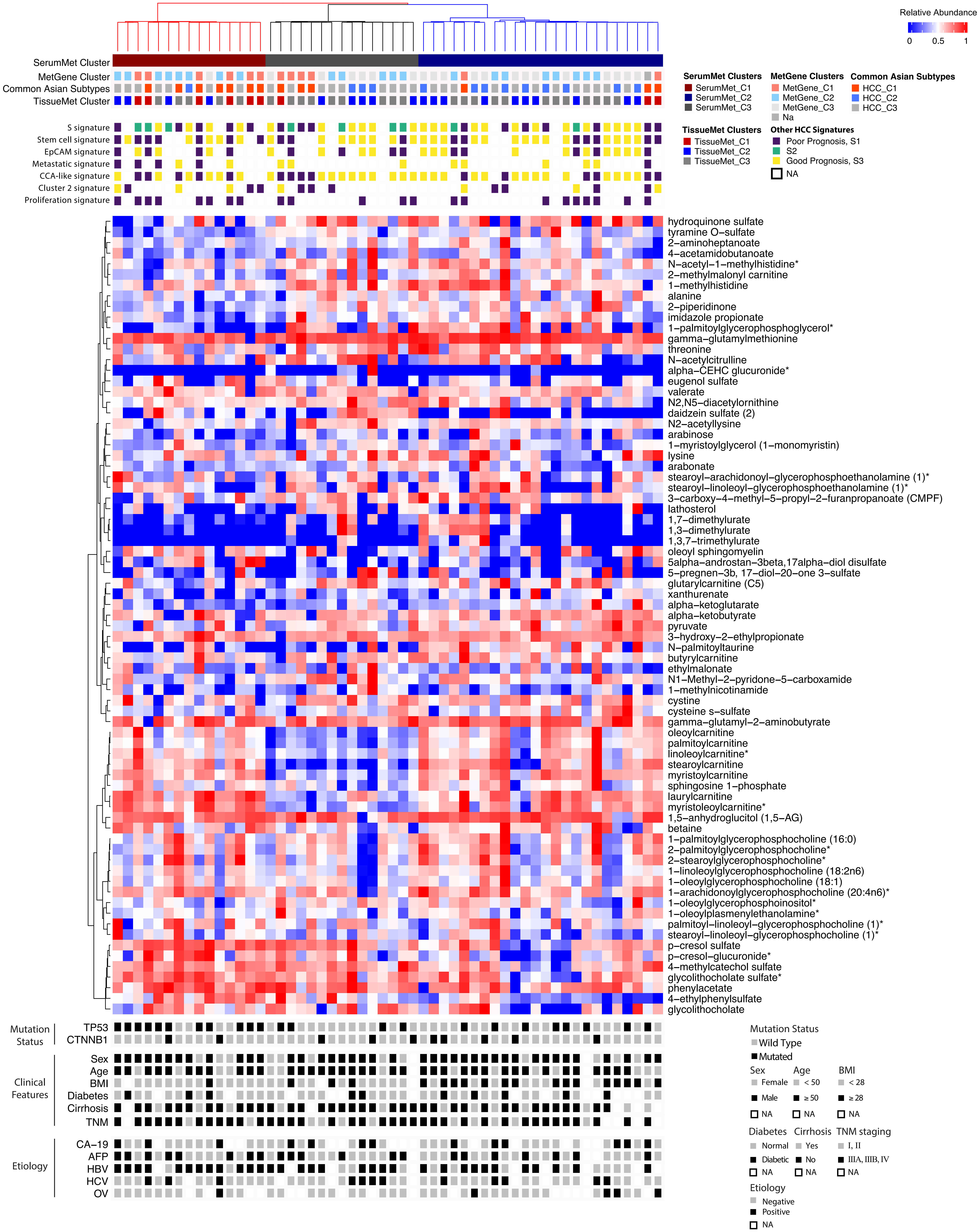

Suppl Fig S7 Altered lipid metabolism networks in HCC tissues and between metabolic gene clusters

A

Lipid Metabolism Network - Tumor/Adjacent Normal Comparison

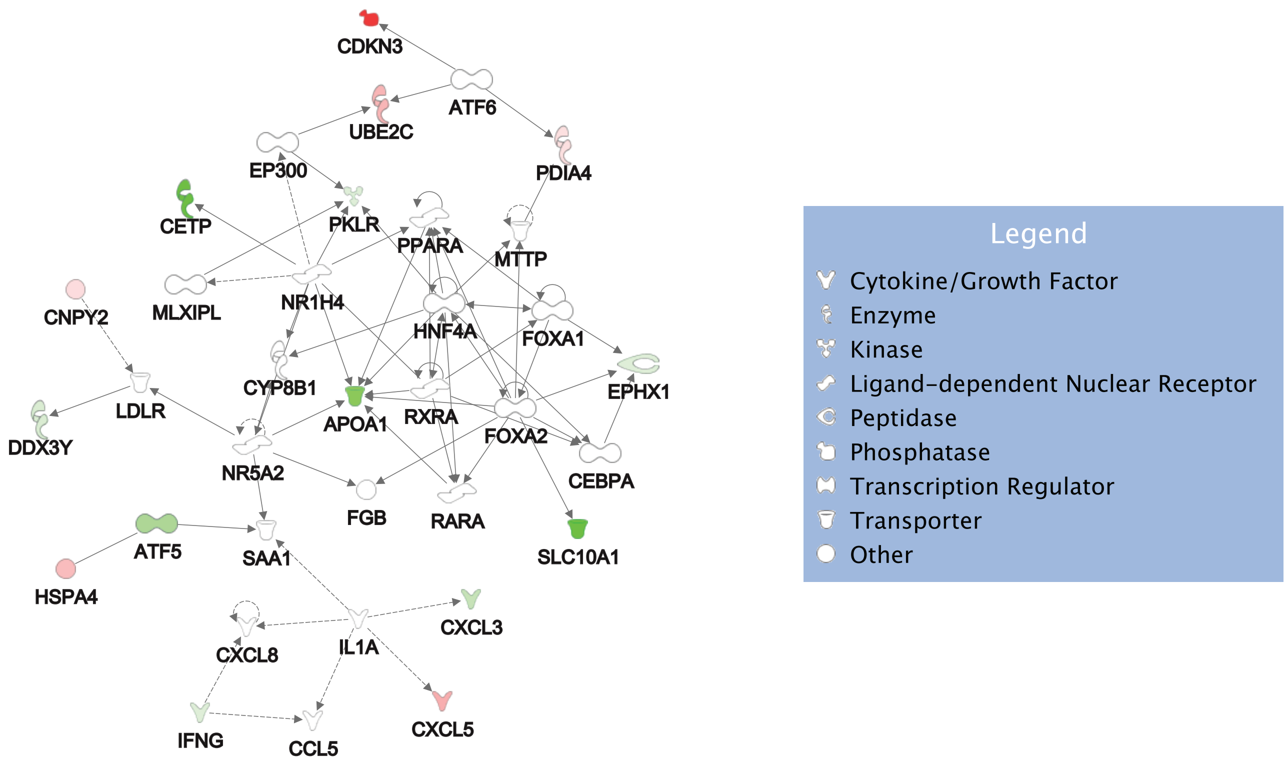

B

Lipid Metabolism Network - MetGene-C1/C2 Comparison

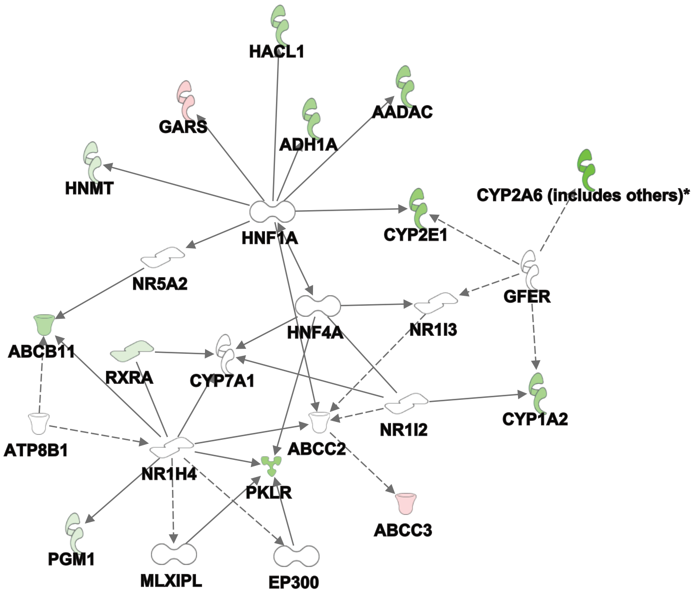

Suppl Fig S8 Gene set enrichment analysis (GSEA) of metaboli genes in tumor and non-tumor tissue

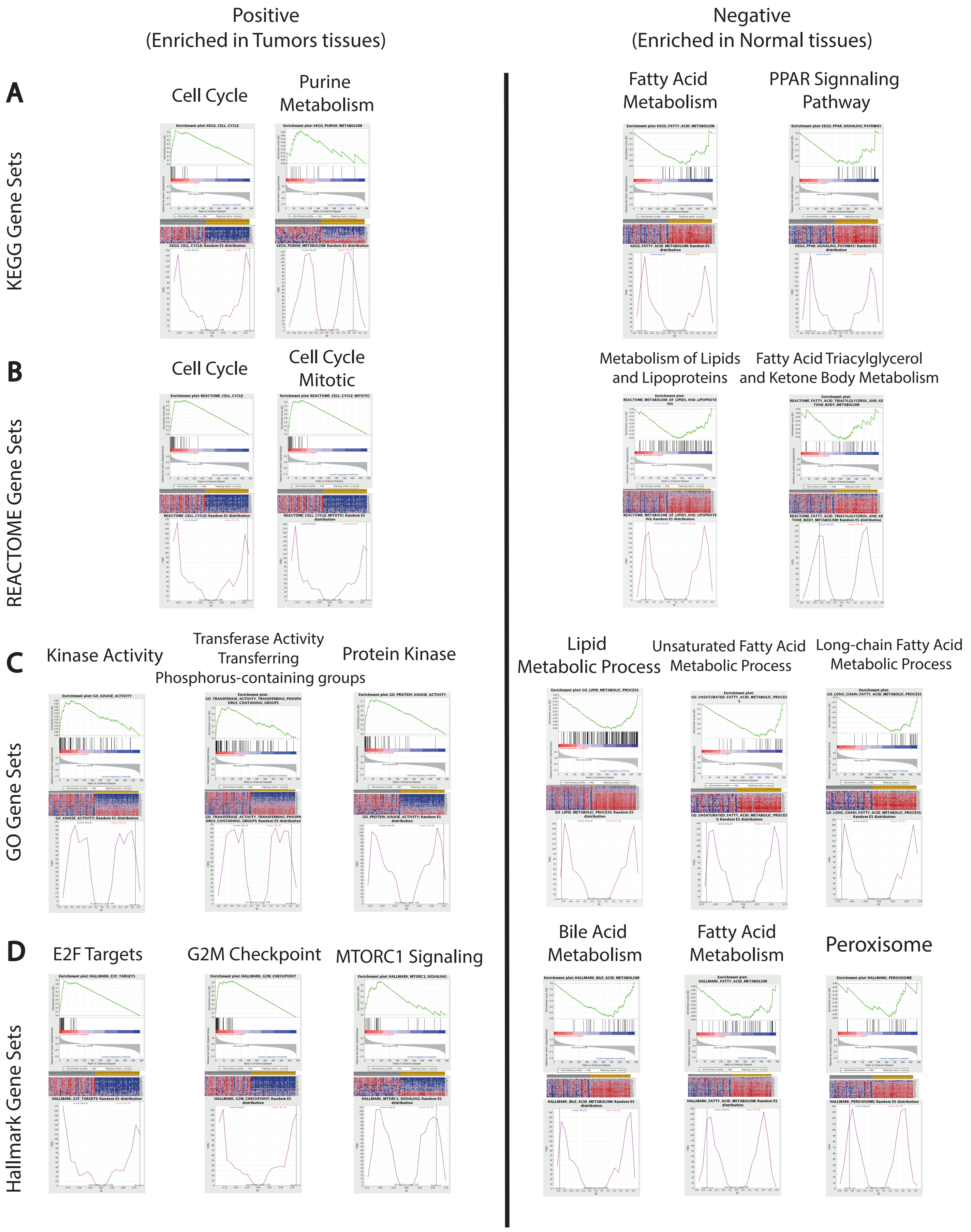

SupplFig S9 Microbial metabolism in diverse environments map

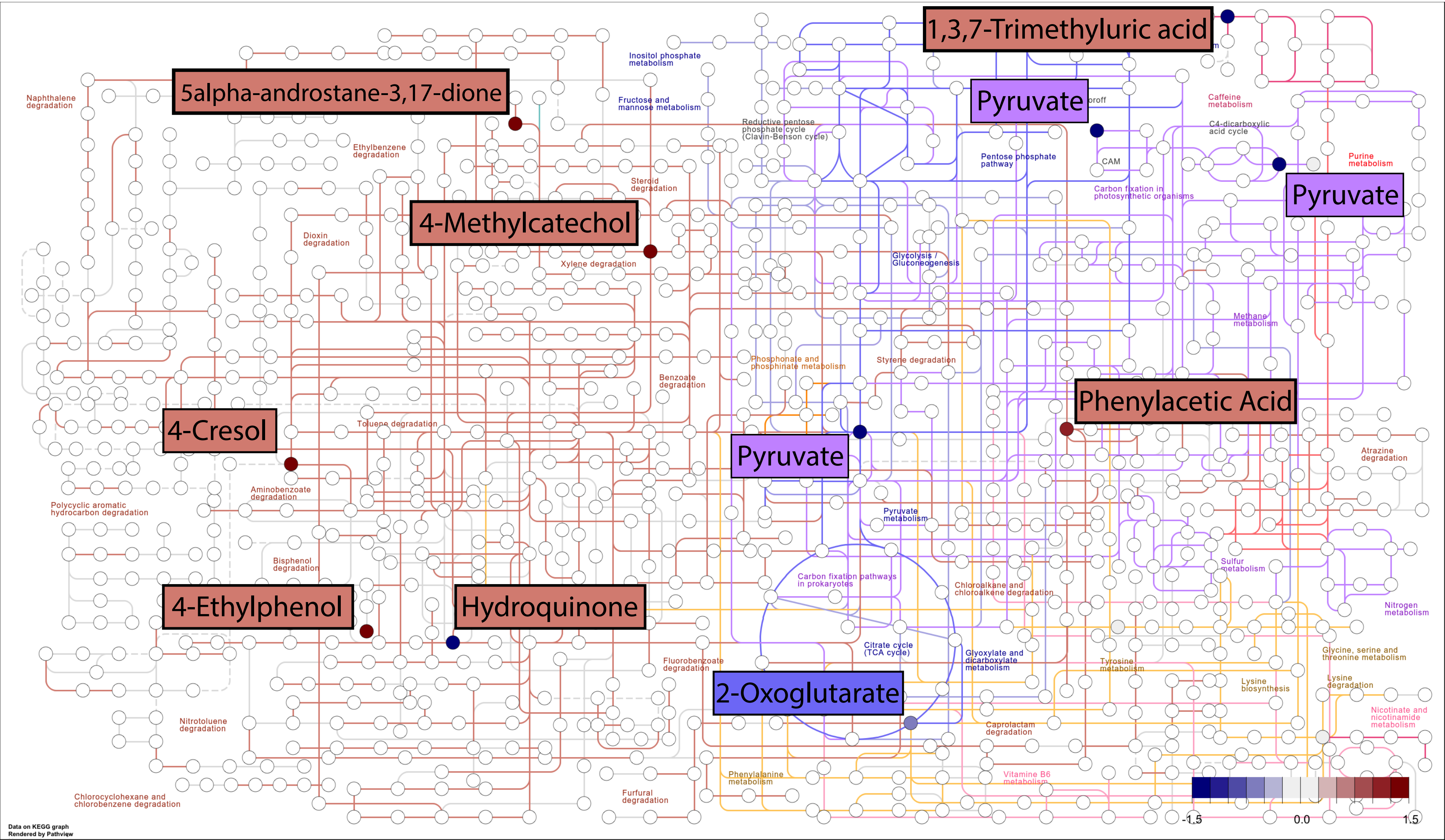

Suppl Fig S10 Permutation of correlation analysis of metabolic genes and tumor tissue metabolites

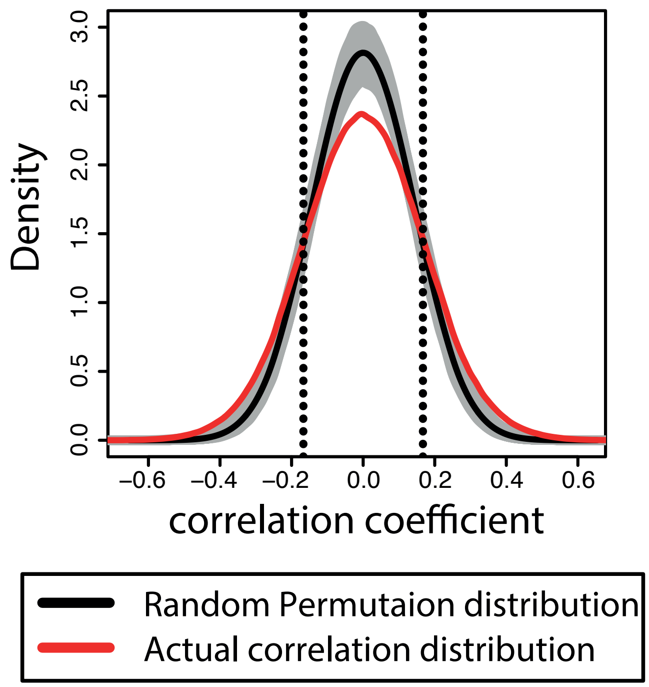

Supplement: Supplementary file 2 — Supplementary Information 2. [file 41598_2021_91560_MOESM2_ESM.pdf]
